# An Osgood Type Regularity Criterion for the 3D Boussinesq Equations

**DOI:** 10.1155/2014/563084

**Published:** 2014-03-11

**Authors:** Qiang Wu, Lin Hu, Guili Liu

**Affiliations:** ^1^School of Resources and Safety Engineering, Central South University, Changsha, Hunan 410075, China; ^2^Jiangxi University of Science and Technology, Ganzhou, Jiangxi 341000, China; ^3^Department of Basic Teaching, Harbin Finance University, Harbin 150030, Heilongjiang, China

## Abstract

We consider the three-dimensional Boussinesq equations, and obtain an Osgood type regularity criterion in terms of the velocity gradient.

## 1. Introduction

In this paper, we consider the following three-dimensional (3D) Boussinesq equations with the incompressibility condition:

(1) $$\begin{array}{c} u_{t}+\left(u\cdot \nabla \right)u-\Delta u+\nabla \pi =\theta e_{3},\\ \theta_{t}+\left(u\cdot \nabla \right)\theta -\Delta \theta =0,\\ \nabla \cdot u=0,\\ u\left(x,0\right)=u_{0},\;\theta \left(x,0\right)=\theta_{0}, \end{array}$$

where **u** = (*u*_1_(*x*, *t*), *u*_2_(*x*, *t*), *u*_3_(*x*, *t*)) is the fluid velocity, *π* = *π*(*x*, *t*) is a scalar pressure, and *θ* = *θ*(*x*, *t*) is the scalar temperature, while **u**_0_ and *θ*_0_ are the prescribed initial velocity and temperature, respectively, with ∇·**u**_0_ = 0.

In case *θ* = 0, (1) reduces to the incompressible Navier-Stokes equations. The regularity of its weak solutions and the existence of global strong solutions are important open problems; see [1–3]. Starting with [4, 5], there have been a lot of literatures devoted to finding sufficient conditions (which now are called regularity criteria) to ensure the smoothness of the solutions; see [6–16] and so forth. Since the convective terms (**u** · ∇)**u** are the same in the Navier-Stokes equations and Boussinesq equations, the authors also consider the regularity conditions for (1). In particular, Qiu et al. [17] obtained Serrin type regularity condition:

(2) $$\begin{array}{c} u\in L^{p}\left(0,T;L^{q}\left({\mathbb{R}}^{3}\right)\right),\;\;\frac{2}{p}+\frac{3}{q}=1,\;3<q⩽\infty . \end{array}$$

The extension to the multiplier spaces was established by the same authors in [18]. For the Besov-type regularity criterion, Fan and Zhou [19] and Ishimura and Morimoto [20] showed the following regularity conditions:

(3) $$\begin{array}{c} \nabla \times u\in L^{1}\left(0,T;{\dot{B}}_{\infty ,\infty }^{0}\left({\mathbb{R}}^{3}\right)\right),\\ \nabla u\in L^{1}\left(0,T;L^{\infty }\left({\mathbb{R}}^{3}\right)\right). \end{array}$$

Zhang [21, 22] then considers the regularity criterion in terms of the pressure or its gradient. The readers are also referred to [23] for generalized models.

Motivated by [24–26], we will improve (3) as in the following.

**Theorem 1**

Let (**u**_0_, *θ*_0_) ∈ *H* ^1^(ℝ^3^). Assume that (**u**, *θ*) is the smooth solution to (1) with the initial data (**u**_0_, *θ*_0_) for 0 ⩽ *t* < *T*. If

(4) $$\begin{array}{c} \underset{2⩽q<\infty }{\sup}\overset{T}{\underset{0}{\int }}\frac{{||{\bar{S}}_{q}\nabla u||}_{L^{\infty }}}{q\ln q}<\infty , \end{array}$$

then the solution (**u**, *θ*) can be extended after time *t* = *T*. Here, ${\dot{\Delta }}_{k}$ denotes the Fourier localization operator and $\Delta S_{q}=\sum_{l=-q}^{q}{\dot{\Delta }}_{l}$.

**Remark 2**

The Osgood type condition (4) is weaker than (3). Notice that, for *q* ∈ [2, *∞*), we have

(5) $$\begin{array}{c} \frac{{||{\bar{S}}_{q}\nabla u||}_{L^{\infty }}}{q\ln q}⩽\frac{1}{q\ln q}\overset{q}{\underset{l=-q}{\sum }}{||{\dot{\Delta }}_{l}(\nabla \times u)||}_{L^{\infty }}⩽C{||\nabla \times u||}_{{\dot{B}}_{\infty ,\infty }^{0}}. \end{array}$$

The rest of this paper is organized as follows. In Section 2, we recall the definition of Besov spaces and some interpolation inequalities.  Section 3 is devoted to proving Theorem 1.

## 2. Preliminaries

Let *𝒮*(ℝ^3^) be the Schwartz class of rapidly decreasing functions. For *f* ∈ *𝒮*(ℝ^3^), its Fourier transform $ℱf=\hat{f}$ is defined by

(6) $$\begin{array}{c} \hat{f}\left(\xi \right)=\int_{{\mathbb{R}}^{3}}f\left(x\right)e^{-ix\cdot \xi }dx. \end{array}$$

Let us choose a nonnegative radial function *φ* ∈ *𝒮*(ℝ^3^) such that

(7) $$\begin{array}{c} 0⩽\hat{\varphi }\left(\xi \right)⩽1,\;\;\hat{\varphi }\left(\xi \right)=\{\begin{array}{cc} 1, & \text{if}\;\;\left|\xi \right|⩽1,\\ 0, & \text{if}\;\;\left|\xi \right|⩾2, \end{array} \end{array}$$

and let

(8) $$\begin{array}{c} \psi \left(x\right)=\varphi \left(x\right)-2^{-3}\varphi \left(\frac{x}{2}\right),\\ \varphi_{j}\left(x\right)=2^{3j}\varphi \left(2^{j}x\right),\;\;\psi_{j}\left(x\right)=2^{3j}\psi \left(2^{j}x\right),\;j\in \mathbb{Z}. \end{array}$$

For *j* ∈ *ℤ*, the Littlewood-Paley projection operators *S*_*j*_ and ${\dot{\Delta }}_{j}$ are, respectively, defined by

(9) $$\begin{array}{c} S_{j}f=\varphi_{j}*f,\;\;{\dot{\Delta }}_{j}f=\psi_{j}*f. \end{array}$$

Observe that ${\dot{\Delta }}_{j}=S_{j}-S_{j-1}$. Also, it is easy to check that if *f* ∈ *L* ^2^(ℝ^3^), then

(10) $$\begin{array}{c} S_{j}f⟶0,\;\text{as}\;\;j⟶-\infty ;\;\;S_{j}f⟶f,\;\text{as}\;\;j⟶+\infty , \end{array}$$

in the *L* ^2^ sense. By telescoping the series, we thus have the following Littlewood-Paley decomposition:

(11) $$\begin{array}{c} f=\overset{+\infty }{\underset{j=-\infty }{\sum }}{\dot{\Delta }}_{j}f, \end{array}$$

for all *f* ∈ *L* ^2^(ℝ^3^), where the summation is the *L* ^2^ sense. Notice that

(12) $$\begin{array}{c} {\dot{\Delta }}_{j}f=\overset{j+2}{\underset{l=j-2}{\sum }}{\dot{\Delta }}_{l}{\dot{\Delta }}_{j}f=\overset{j+2}{\underset{l=j-2}{\sum }}\psi_{l}*\psi_{j}*f; \end{array}$$

then from Young's inequality, it readily follows that

(13) $$\begin{array}{c} {||{\dot{\Delta }}_{j}f||}_{L^{q}}⩽C2^{3j(1/p-1/q)}{||{\dot{\Delta }}_{j}f||}_{L^{p}}, \end{array}$$

where 1 ⩽ *p* ⩽ *q* ⩽ *∞* and *C* is an absolute constant independent of *f* and *j*.

Let −*∞* < *s* < *∞*, 1 ⩽ *p*, *q* ⩽ *∞*; the homogeneous Besov space ${\dot{B}}_{p,q}^{s}$ is defined by the full-dyadic decomposition such that

(14) $$\begin{array}{c} {\dot{B}}_{p,q}^{s}=\left\{f\in {𝒵}^{\prime }\left({\mathbb{R}}^{3}\right);{||f||}_{{\dot{B}}_{p,q}^{s}}<\infty \right\}, \end{array}$$

where

(15) $$\begin{array}{c} {||f||}_{{\dot{B}}_{p,q}^{s}}={||{\left\{2^{js}{||{\dot{\Delta }}_{j}f||}_{L^{p}}\right\}}_{j=-\infty }^{+\infty }||}_{\ell^{q}}, \end{array}$$

and *𝒵*′(ℝ^3^) is the dual space of

(16) $$\begin{array}{c} 𝒵\left({\mathbb{R}}^{3}\right)=\left\{f\in 𝒮\left({\mathbb{R}}^{3}\right);\;\;D^{\alpha }\hat{f}\left(0\right)=0,\;\forall \alpha \in {\mathbb{N}}^{3}\right\}. \end{array}$$

Also, it is well known that

(17) $$\begin{array}{c} {\dot{H}}^{s}\left({\mathbb{R}}^{3}\right)={\dot{B}}_{2,2}^{s}\left({\mathbb{R}}^{3}\right),\;\forall s\in \mathbb{R}. \end{array}$$

We refer to [27] for more detailed properties.

## 3. Proof of Theorem 1

This section is devoted to proving Theorem 1. From standard continuity arguments, we need to only provide the uniform *H* ^1^ bounds of the solution (**u**, *θ*).

Taking the inner products of (1)_1_ with −Δ**u**, (1)_2_ with −Δ*θ*, we obtain by adding together that

(18) 12ddt||∇(u,θ)||L22+||Δ(u,θ)||L22 =∫ℝ3[(u·∇)u]·Δu dx−∫ℝ3θΔu3 dx  +∫ℝ3[(u·∇)θ]·Δθ dx =∫ℝ3∂kθ∂ku3 dx  −∫ℝ3∂kuj(∂jui∂kui+∂jθ∂kθ)dx ≡I+J.

For *I*, we use Hölder's inequality to get

(19) $$\begin{array}{c} I_{1}⩽\frac{1}{2}{||\nabla (u,\theta )||}_{L^{2}}^{2}. \end{array}$$

For *J*, applying the Littilewood-Paley decomposition as in (11), we get

(20) $$\begin{array}{c} \nabla u=\underset{l<-q}{\sum }\dot{\Delta }\nabla u+\overset{q}{\underset{l=-q}{\sum }}\dot{\Delta }\nabla u+\underset{l>q}{\sum }\dot{\Delta }\nabla u, \end{array}$$

where *q* is positive integral to be determined later on. Plugging (20) into *J*, we see that

(21) J⩽∑l<−q∫ℝ3|Δ˙l∇u|‍‍·|∇(u,θ)|2 dx +∫ℝ3|∑l=−qqΔ˙l∇u‍|‍·|∇(u,θ)|2 dx +∑l>q∫ℝ3|Δ˙l∇u|‍‍·|∇(u,θ)|2 dx≡J1+J2+J3.

For *J*_1_, we dominate as

(22) J1⩽∑l<−q||Δ˙l∇u||L∞‍||∇(u,θ)||L22⩽C∑l<−q23l/2||Δ˙l∇u||L2‍||∇(u,θ)||L22 (by  (13))⩽C(∑l<−q2(3l/2)·2‍)1/2·(∑l<−q ||Δ˙l∇u||L22‍)1/2||∇(u,θ)||L22⩽C2−3q/2||∇u||L2||∇(u,θ)||L22 (by  (17))=[C2−q/2||∇(u,θ)||L2]3.

For *J*_2_, we have

(23) J2=∫ℝ3|S¯q∇u|‍·|∇(u,θ)|2 dx⩽||S¯q∇u||L∞||∇(u,θ)||L22.

Finally, for *J*_3_, we estimate as

(24) J3⩽∑l>q||Δl∇u||L3‍||∇(u,θ)||L32⩽C ∑l>q2l/2||Δl∇u||L2‍||∇(u,θ)||L2||Δ(u,θ)||L2 (by  (13)  and  Gagliardo-Nireberg  inequality)⩽C(∑l>q2−(l/2)·2‍)1/2·(∑l>q2l·2||Δ˙l∇u||L22‍)1/2 ×||∇(u,θ)||L2||Δ(u,θ)||L2⩽[C2−q/2||∇(u,θ)||L2]||Δ(u,θ)||L22 (by  (17)).

Gathering (22), (23), and (24) together and plugging them into (21), we deduce

(25) J⩽[C2−q/2||∇(u,θ)||L2]3+||S¯q∇u||L∞||∇(u,θ)||L22+[C2−q/2||∇(u,θ)||L2]||Δ(u,θ)||L22.

Substituting (19) and (25) into (18), we find

(26) 12ddt||∇(u,θ)||L22+||Δ(u,θ)||L22 ⩽12||∇(u,θ)||L22+[C2−q/2||∇(u,θ)||L2]3  +||S¯q∇u||L∞qln⁡q·qln⁡q||∇(u,θ)||L22  +[C2−q/2||∇(u,θ)||L2]||Δ(u,θ)||L22.

Taking

(27) $$\begin{array}{c} q=\left[\frac{2}{\ln\;2\;\ln^{+}\left(C{||\nabla (u,\theta )||}_{L^{2}}\right)}\right]+1, \end{array}$$

where [*t*] is the largest integer smaller than *t* ∈ ℝ and ln⁡^+^ *t* = ln⁡(*e* + *t*), then (26) implies that

(28) ddt||∇(u,θ)||L22 ⩽||∇(u,θ)||L22+C  +||S¯q∇u||L∞qln⁡qln⁡+(||∇(u,θ)||L2)ln⁡+ln⁡+(||∇(u,θ)||L2)  ×||∇(u,θ)||L22.

Applying Gronwall inequality three times, we deduce

(29) ||∇(u,θ)||L22+∫0t||Δ(u,θ)||L2 dτ ⩽C exp⁡ exp exp(∫0t||S¯q∇u||L∞qln⁡q dτ).

Recalling (4), we see the solution (**u**, *θ*) is uniformly bounded in *L* ^*∞*^(0, *T*; *H* ^1^(ℝ^3^)). This completes the proof of Theorem 1.
